# Supplementary material for: A Novel One-Step Reactive Extrusion Process for High-Performance Rigid Crosslinked PVC Composite Fabrication Using Triazine Crosslinking Agent@Melamine-Formaldehyde Microcapsules
Source: Materials (Basel). 2023 Jun 26;16(13):4600. doi: 10.3390/ma16134600 (PMC10342326; doi:10.3390/ma16134600)
Supplement: Supplementary file 1 [file materials-16-04600-s001.zip › materials-2415778-supplementary.pdf]

materials

MDPI

Supporting Information

A Novel One-Step Reactive Extrusion Process for High-Performance Rigid Crosslinked PVC Composite Fabrication Using Triazine Crosslinking Agent@Melamine-Formaldehyde Microcapsules

Table S1. Different preparation formulas and performance of DB@MF.

Parameter	DB@MF-1	DB@MF-2	DB@MF-3
Formaldehyde (37 wt%) (g)	3.000	3.500	4.000
Melamine (g)	1.875	2.188	2.500
DB (g)	3.200	3.200	3.200
SDBS (g)	0.4500	0.4500	0.4500
Core content (%) [1]	58.697	46.912	45.866
Young's modulus (MPa) [2]	3677	732	30071

[1] Calculated by formula (1).
[2] Simulated by nanoscope analysis software.

Table S2. XPS and EA data for different DB@MF.

Sample	XPS				EA			
	C_{XPS} (%)	N_{XPS} (%)	O_{XPS} (%)	S_{XPS} (%)	C_{EA} (%)	H_{EA} (%)	N_{EA} (%)	S_{EA} (%)
DB@MF1	59.53	32.67	3.42	4.38	48.31	7.625	26.66	13.81
DB@MF2	52.52	41.14	4.02	2.32	44.59	6.981	31.80	11.04
DB@MF3	51.23	42.40	4.50	1.87	44.78	7.102	31.85	10.79

Table S3. Formulation of the DB@MF/PVC composites.

Parameter	Score (phr)
PVC	100
Calcium-zinc Stabilizer	8.54
DB [1]	*a*
Magnesium Oxide	2*a*

[1] The actual addition of DB@MF was calculated by the core content.

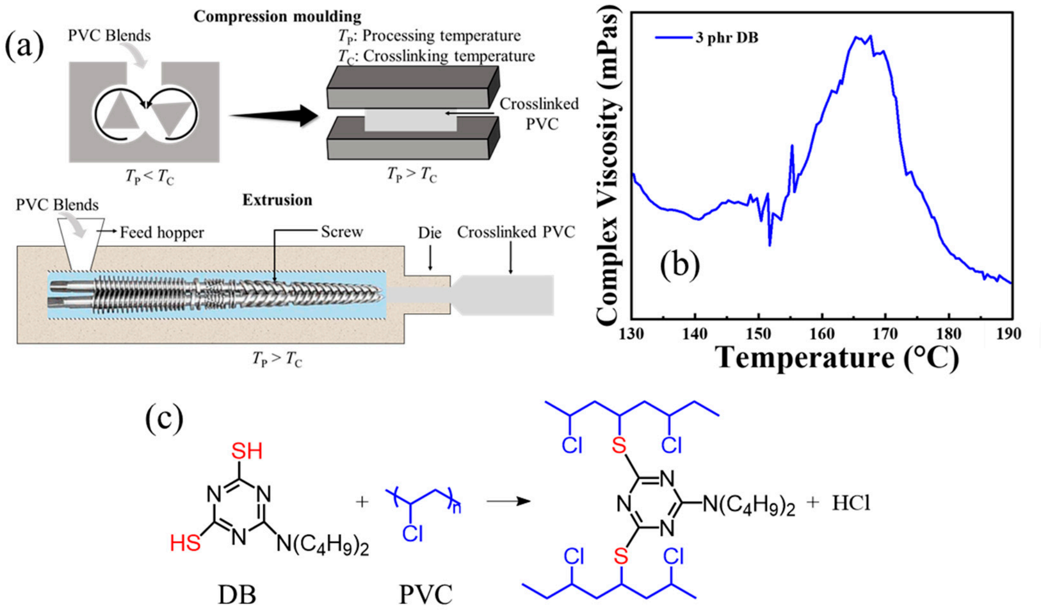

Figure S1. (**a**) PVC processing method. (**b**) The rotational rheometer curve of PVC with pure DB. (**c**) The process of the PVC chemical crosslinking reaction.

For the preparation of the samples for the rotational rheometer test using two-step compression moulding, PVC was plasticized with pure DB at 130°C by a torque rheometer, and PVC composites were subsequently moulded at 130°C. The curve in Figure S1b shows that the temperature used to initiate the crosslinking reaction was 153°C lower than the PVC processing temperature.

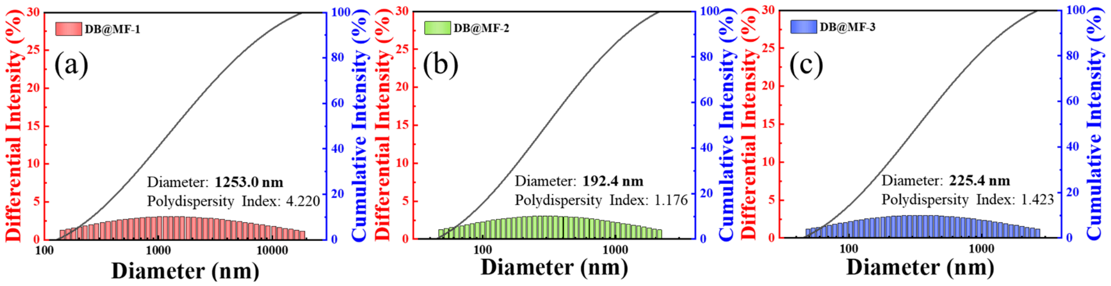

Figure S2. Particle size distribution of different DB@MFs.

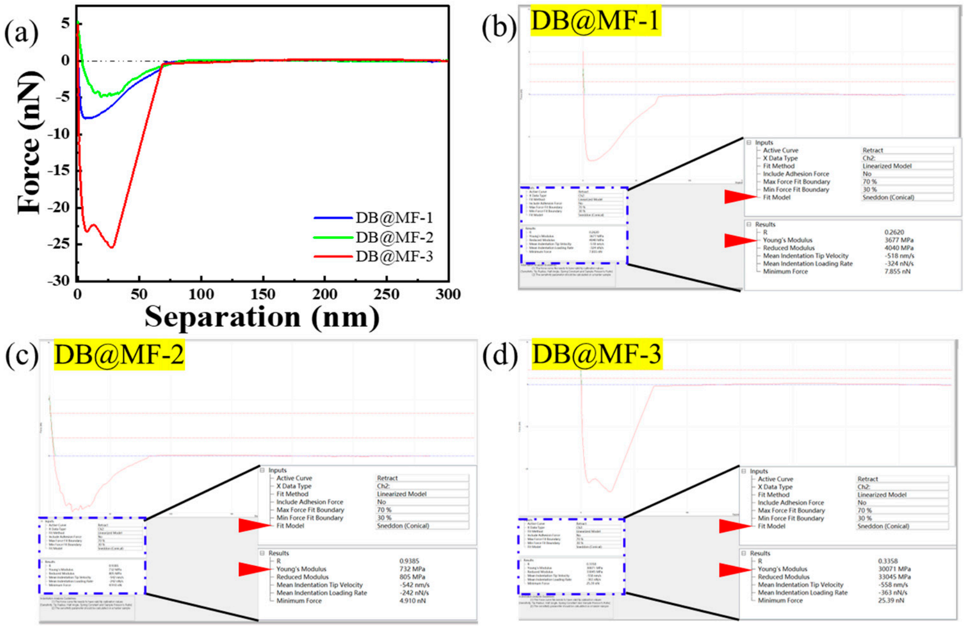

Figure S3. (**a**) Force–separation curves of different DB@MFs. (**b–d**) The simulation results for different curves were developed with Nanoscope Analysis Software.

To determine the Young's modulus of the microcapsule shell material, a small amount of a dried microcapsule sample was placed on a glass slide, and then the top of the microcapsule in the same area was scanned with a conical silicon cantilever in intermittent contact mode to obtain the force–displacement curve (Figure S3a). The obtained force–displacement curves were then analyzed and simulated using NanoScope analysis software (Figure S3b, c and d), which allowed us to obtain the Young's modulus of the microcapsule shell material.

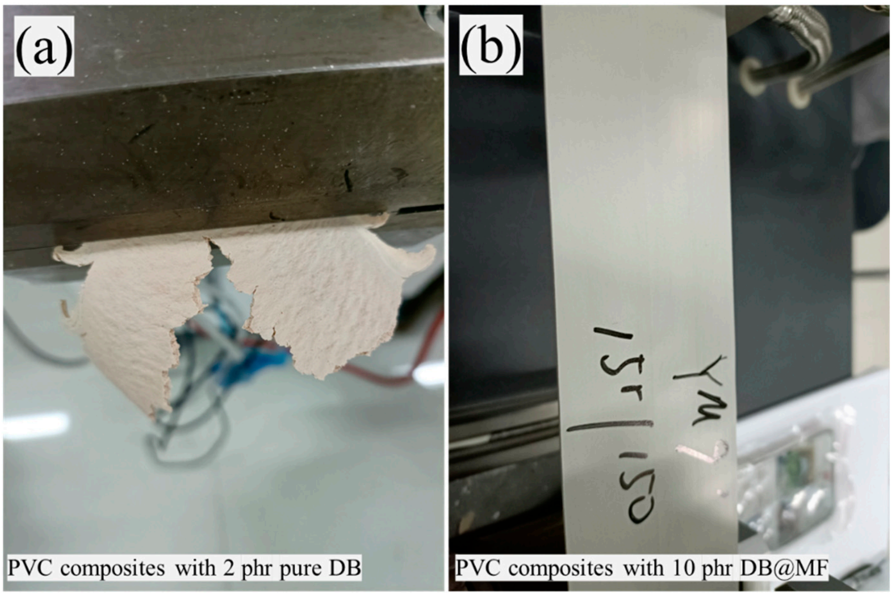

Figure S4. The twin screw extrusion of (**a**) PVC composites with 2 phr pure DB, (**b**) 10 phr.

Figure S4a shows the unencapsulated crosslinking agents mixed with PVC, which could not be extruded continuously due to premature crosslinking. Figure S4b shows the encapsulated crosslinking agents mixed with PVC. The crosslinked PVC was smoothly extruded.

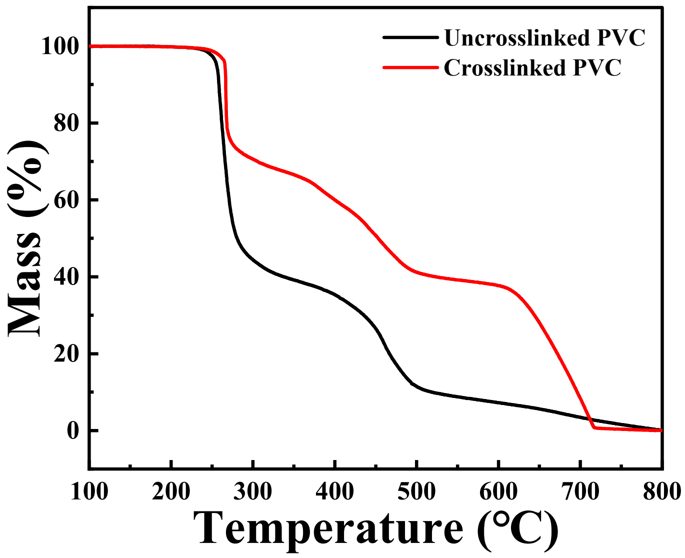

Figure S5. Heat weight loss curves of PVC composites.

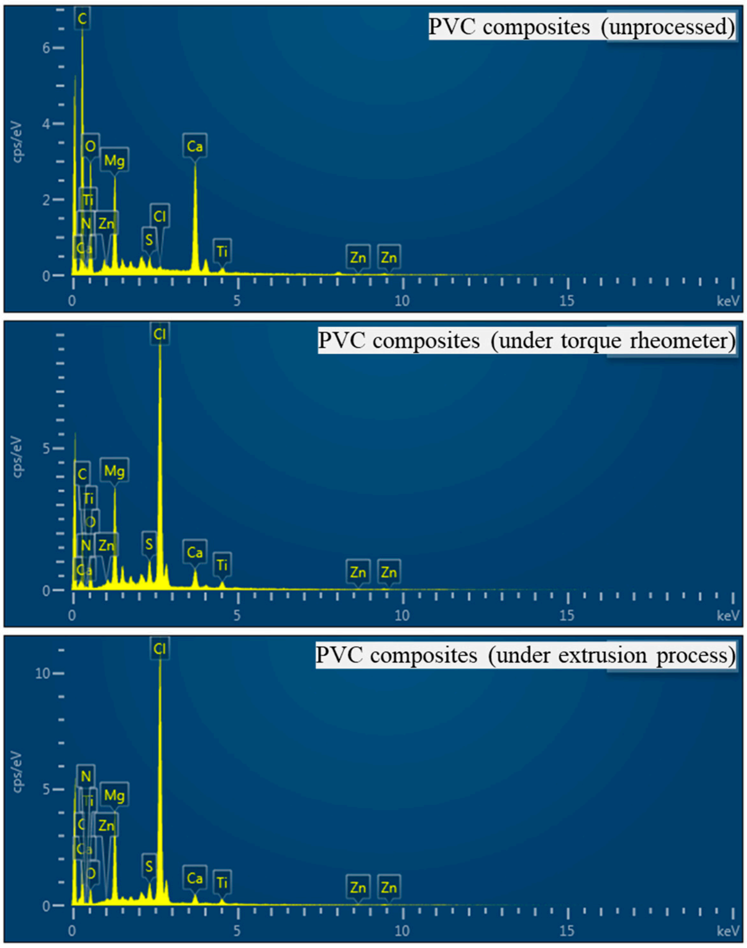

Figure S6. SEM-EDX mapping of PVC composites before and after processing.
